# Facile Synthesis of Carbon Nanosphere/NiCo_2_O_4_ Core-shell Sub-microspheres for High Performance Supercapacitor

**DOI:** 10.1038/srep12903

**Published:** 2015-08-06

**Authors:** Delong Li, Youning Gong, Yupeng Zhang, Chengzhi Luo, Weiping Li, Qiang Fu, Chunxu Pan

**Affiliations:** 1 Shenzhen Research Institute, Wuhan University, Shenzhen 518057, China; 2 School of Physics and Technology, Wuhan University, Wuhan 430072, China; 3 Department of Materials Engineering, Monash University, Victoria 3800, Australia

## Abstract

This paper introduced a process to prepare the carbon nanosphere (CNS)/NiCo_2_O_4_ core-shell sub-microspheres. That is: 1) CNSs were firstly prepared via a simple hydrothermal method; 2) a layer of NiCo_2_O_4_ precursor was coated on the CNS surface; 3) finally the composite was annealed at 350 °C for 2 hours in the air, and the CNS/NiCo_2_O_4_ core-shell sub-microspheres were obtained. This core-shell sub-microsphere was prepared with a simple, economical and environmental-friendly hydrothermal method, and was suitable for large-scale production, which expects a promising electrode candidate for high performance energy storage applications. Electrochemical experiments revealed that the composite exhibited remarkable electrochemical performances with high capacitance and desirable cycle life at high rates, such as: 1) the maximum specific capacitance was up to 1420 F/g at 1 A/g; 2) about 98.5% of the capacitance retained after 3000 charge-discharge cycles; 3) the capacitance retention was about 72% as the current density increase from 1 A/g to 10 A/g.

In the past few years, supercapacitors (SCs) have attracted considerable attention due to their high power density, long life cycle, and fast recharge capability. In general, the electrode materials for supercapacitors can be divided into two categories on the basis of the energy storage mechanism: electrical double layer capacitors (EDLCs) and pseudo-capacitors^1^. However, pseudo-capacitors exhibit much larger capacitance values and energy density than EDLCs due to that the pseudo-capacitive materials taking full advantages of the fast and reversible redox reactions of the electrochemically active electrode materials^2^,^3^.

It is well-known that transition metal oxides are of ultra high pseudoactive capacitance^4^,^5^,^6^. Recently, mixed transition-metal oxides (MTMOs), such as single-phase ternary metal oxides with two different metal cations, typically in a spinel structure A_x_B_3-x_O_4_ (A, B = Co, Ni, Zn, Mn, Fe, and so on), have captured much attentions as promising electrode materials in electrochemical energy conversion and storage systems^7^,^8^,^9^. Due to complex chemical compositions and relatively low activation energy for electron transfer between cations, MTMOs exhibit the higher electrochemical activity and electrical conductivity than the simple transition-metal oxides^7^,^9^.

Among the MTMOs, the spinel nickel cobaltite (NiCo_2_O_4_) is one of the promising electrode material, which shows many outstanding advantages including high theoretical capacitance (higher than 3000 F/g), low cost, natural abundance and environmental-friendly^10^,^11^. NiCo_2_O_4_ holds great promise as a supercapacitor electrode material owing to its high specific capacitance and rate capabilities^12^,^13^. However, due to relatively poor conductivity and limited specific area, the practical capacitance of NiCo_2_O_4_ is much lower than the theoretical capacitance value^2^.

In order to overcome these disadvantages, many studies have been done to optimization of morphology and incorporating NiCo_2_O_4_ with conductive materials for obtaining a high specific capacitance^2^,^4^,^14^,^15^,^16^,^17^. Cai *et al.*^2^ prepared the CNT@NiCo_2_O_4_ core-shell structural nanocable by a facile chemical co-deposition route, and the as-prepared CNT@NiCo_2_O_4_ nanocables exhibited a high capacitance of 1038 F/g at a current density of 0.5 A/g. Deng *et al.*^4^ synthesized ultrathin mesoporous NiCo_2_O_4_ nanosheets on carbon fiber paper, which showed a high specific capacitance and desirable cycling stability, due to the contributions involving high porosity and good electric conductivity of the CFP substrate. Luo *et al.*^15^ fabricated irregular porous network-like NiCo_2_O_4_-reduced graphene oxide (rGO) nanocomposite, and the rGO served as a conductive network to facilitate the collection and transportation of electrons during the cycling.

As many kinds of carbon materials have been proven to be excellent electrode materials, including graphene, carbon nanotubes, porous carbon and so on^18^,^19^,^20^,^21^, carbon materials usually are used for improving the electrochemistry performance of metal oxides. In this paper, we reported a novel route to synthesis CNS/NiCo_2_O_4_ core-shell structural sub-microspheres, in which the CNS act as a core and NiCo_2_O_4_ coated on the CNS surface, which exhibited a high specific capacitance and excellent cycling stabilities at high current density. It is of advantages including simple, economical, environmental-friendly, and mass production, which exhibits a potential industrial application for high performance supercapacitors as an electrode material.

The phase structures of the samples were studied by powder X-ray diffraction (XRD). Figure 1a illustrates the XRD patterns of the CNS, NiCo_2_O_4_ and CNS/NiCo_2_O_4_. The diffraction peaks at 2 theta = 18.91°, 31.12°, 36.71°, 38.41°, 44.57°, 55.52°, 59.20°, and 64.92° are indexed as the crystal planes (111), (220), (331), (222), (400), (422), (511), and (440) of NiCo_2_O_4_. The crystallite size of NiCo_2_O_4_ is estimated base on XRD pattern. According to the Scherrer equation, the average crystallite size of NiCo_2_O_4_ is about 17.9 nm. In the XRD pattern of CNS/NiCo_2_O_4_, in addition to the pronounced peaks of spinel phase NiCo_2_O_4_, the diffraction peak of CNSs can not be identified clearly due to the mass ratio of CNSs in the composite was too low. Actually the mass ratio of CNS in the composite is roughly about 5.3% (as shown in Fig. S1 in Supplementary information).

However, the existence of CNS can be proven by Raman spectroscopy. As shown in Fig. 1b, the peaks at 151.8, 457.8, 455.2, 505.7, 656.7 and 1096.7 cm^−1^ correspond to the F_2g_, E_g_, L_O_, A_1g_ and 2L_O_ modes of NiCo_2_O_4_, respectively, while the peaks at 1368.5 and 1593.3 cm^−1^ correspond to the D and G band of carbon. These results are well consistent with previously reported literatures^22^,^23^.

The species and chemical states of elements in the surface of the composite materials were analysis by XPS. Obviously, the CNS/NiCo_2_O_4_ composite had predominant C1s, O1s, Ni2p and Co2p peaks (as shown in Fig. S2 in supplementary information). Figure 2 illustrates the XPS spectra of the CNS/NiCo_2_O_4_ composite. The following messages were obtained: 1) Figure 2a shows two main peaks, which correspond to the SP2 carbon (C1, ~284.8 eV) and C-OH (C2, ~285.6 eV)^18^,^20^,^24^,^25^. While the weak fitting peak at binding energy of 287.2 eV (C3) was ascribed to C-O bond^18^. 2) As shown in Fig. 2b, O1s peak also could be divided into three different peaks (marked as O1, O2, and O3), which corresponded to the metal oxygen bonds (O1, ~529.5 eV), oxygen ions (O2, ~531.2 eV) and physic or chemisorbed water at or within the surface (O3, ~533.1 eV)^26^,^27^,^28^,^29^. O 1s spectra at binding energies of 529.5 and 531.2 eV were ascribed to O^2−^ species in NiCo_2_O_4_; 3) By using Gaussian fitting, the Ni 2p spectrum (Fig. 2c) is fitted considering two spin-orbit doublets characteristic of Ni^2+^ and Ni^3+^ and two shakeup satellites^26^,^29^. According to the fitting data, the fitting peaks at binding energy of 855.7 and 873.7 eV are indexed to Ni^2+^, while the fitting peaks at binding energy of 854.1 and 872.0 eV are ascribed to Ni^3+^, respectively; 4) In Co 2p spectrum(Fig. 2d), two kinds of Co species are also observed. The fitting peaks at binding energies of 781.4 eV and 796.2 eV are indexed to Co^2+^, while the other two fitting peaks at binding energies of 779.7 eV and 794.7 eV belong to Co^3+^. Also, the Co^3+^/Co^2+^ were also coexisting in the core-shell composite. These results were consistent with the previous reports for NiCo_2_O_4_^29^,^30^, and further confirmed the coexistence of CNS and NiCo_2_O_4_ in the composite. In addition, the existence of cations Co^3+^/Co^2+^ and Ni^3+^/Ni^2+^ in the CNS/NiCo_2_O_4_ composite provided abundant active sites for energy storage.

Figure 3 shows the SEM morphologies of the CNS/NiCo_2_O_4_ composite. It could be seen that the CNS/NiCo_2_O_4_ composite was in typically spheres with rough surface, and the diameters were in a range of 250 ~ 300 nm. Figure 4a,b shows the HRTEM image of the CNS/NiCo_2_O_4_ spheres. Clearly, a core-shell structure was observed, which showed a CNS was a core and NiCo_2_O_4_ was coated on the surface. The lattice fringe of the coated layer revealed that with interplanar distance of 0.47 nm corresponded to the (111) planes of spinel-structured NiCo_2_O_4_. However, the crystalline structure of the CNSs could not be directly observed due to the NiCo_2_O_4_ coated layer.

According to TEM observations, the shell was composed of numerous NiCo_2_O_4_ nanoparticles with controlled size and compositions. This kind of structure shows advantages in facile penetration of liquid electrolyte and effective buffering of large volume changes during charge/discharge process^31^. The corresponding Fast Fourier Transformation (FFT) image was shown in Fig. 4d, which demonstrates polycrystalline of the sub-microspheres.

Figure 4f,e shows the EDS elemental maps of the CNS/NiCo_2_O_4_ core-shell sub-microspheres. Clearly, the elements oxygen (O), nickel (Ni), cobalt (Co) and carbon(C) were well distributed in the core-shell sub-microspheres.

The electrochemical properties of the CNS/NiCo_2_O_4_ core-shell sub-microspheres were measured by using various techniques involving cyclic voltammetry (CV), galvanostatic charge/discharge (GCD) curves and EIS in a three electrode system. Figure 5a illustrates the CV curves with variant scanning rates ranging from 5 to 100 mV/s. According to experimental results, a pair of redox current peaks was existed in all CV curves. Generally, the redox couples correspond to the reversible reactions of M-O/M-O-OH (M represent Ni or Co)^8^. With the sweep rate rising from 5 mV to 100 mV, the position of the cathodic peak shifted from 0.26 V to 0.107 V, which indicated a low resistance of the electrodes^4^.

Figure 5b gives the GCD curves at different current densities of the CNS/NiCo_2_O_4_ core-shell sub-microspheres. Because the redox reaction between Ni/Co cations and OH anions is a diffusion-controlled process through electrode grain boundaries^32^, therefore, the specific capacitance decreases as the current density increases. Figure 5c is the corresponding specific capacitance as a function of current density. That is to say, when the discharge current densities were at 1 A/g and 10 A/g, the specific capacitances were 1420 F/g and 1018 F/g, respectively. Over the current density range, the specific capacitance decreased to 71.7% of its initial value. Comparing with reported data^10^,^33^, the present results exhibited the same or better rate performance. This might attributed to the unique hierarchical core-shell structure, which provided massive electroactive sites^34^, and the CNSs facilitated the electron transport during electrochemical reaction. Moreover, in addition to the conductivity, CNSs could also effectively prevent the agglomeration of NiCo_2_O_4_ and ensured the full utilization of electroactive materials.

The cyclability of the CNS/NiCo_2_O_4_ core-shell sub-microspheres electrode was evaluated by the repeated GCD measurement at current density of 10 A/g, as shown in Fig. 5d. Obviously, the specific capacitance of the CNS/NiCo_2_O_4_ core-shell sub-microspheres slightly decrease to 98.5% for the first cycle after 3000 time’s test, which indicated its excellent cycling stability.

In general, EIS was usually used to investigate the performance of electrochemical capacitors, such as internal resistance, capacity, etc. The EIS data were analyzed by using Nyquist plots, which showed the frequency response of the electrode/electrolyte system and were the plots of the imaginary component (Z”) of the impedance against the real component (Z’)^35^. As shown in Fig. 6, the Nyquist plot of the CNS/NiCo_2_O_4_ electrode is presented with the equivalent circuit inset. In the high frequency region, the CNS/NiCo_2_O_4_ core-shell sub-microspheres showed inconspicuous loop from an expanded view, which indicated a minimal charge-transfer resistance between the electroactive materials and the electrolyte interface. The curve of the lower frequency showed the impedance of the electroactive materials, which was mostly caused by the ion diffusion within the electroactive materials^36^. At high frequency, the intercept on real axis represents a combined resistance (Rs) containing intrinsic resistance of electrode materials, ionic resistance of electrolyte and contact resistance between electrode and current collector. The EIS plot exhibits identical Rs value about 3.25 Ω and 3.54 Ω before and after 3000 cycles of the charging/discharging experiments. Obviously, a quasi-semicircle was observed at higher frequency range and its diameter corresponded to the charge transfer resistance (Rct) caused by Faradic reactions. The fitted value of Rct obtained for electrode was about 4.01 Ω and 4.05 Ω before and after 3000 cycles of the charging/discharging experiments. However, a minimal slope differences was observed from the vertical diffusion lines, indicating the excellent capacitive performance of the electrode before and after 3000 cycles of the charging/discharging experiments. These results revealed good stability of the CNS/NiCo_2_O_4_ core-shell sub-microspheres.

In summary, compared to single component NiO and Co_3_O_4_, NiCo_2_O_4_ is a promising electrode material, due to its outstanding advantages including high theoretical capacitance, low cost, natural abundance and environmental-friendly. However, its relatively poor conductivity and small specific area limited its practical capacitance much lower than the theoretical value. In this work, CNS/NiCo_2_O_4_ core-shell sub-microspheres were successfully prepared via a facile and simple hydrothermal process.

The experimental results revealed that this composite exhibited a remarkable capacitance performance, when it was used as an electrode material. The reason is as follows: 1) the conductive CNSs facilitate the electron transport; 2) the unique hierarchical core-shell structure provides massive electroactive sites; 3) CNSs effectively prevent the NiCo_2_O_4_ agglomeration and ensure the full utilization of the electroactive materials. The CNS/NiCo_2_O_4_ core-shell sub-microspheres is suitable for large-scale synthesis with a simple, economical and environmental-friendly hydrothermal method, which shows a potential applications in area of supercapacitors, Li-ion battery, etc.

## Methods

Carbon nanospheres (CNSs) were prepared via a simple hydrothermal method, i.e., 1) 0.5 g glucose was added into 20 ml deionized water and transferred to a 25 ml Teflon-lined stainless autoclave; 2) The autoclave heated with hydrothermal condition of 200 °C for 24 hours; 3) After it was cooled down to room temperature, the products were cleaned for several times with DI water and ethanol, and finally dried at 60 °C for 4 hours. In order to acknowledge high crystallization CNSs, the producte was annealed at 400 °C for 4 hours in Ar.

The CNS/NiCo_2_O_4_ core-shell sub-microspheres were prepared according to the process: 1) 10 mg CNSs was mixed with 10 mg/ml sodium oleate methanol solution; 2) After the CNSs were uniformly dispersed by ultrasonication for 1 hour, the resultant solution was mixed with 1 g urea, 2 mmol Co(NO_3_)_2_·6 H_2_O and 1 mmol Ni(NO_3_)_2_·6 H_2_O; 3) The solution was stirred for 2 hours at 60 °C to form a uniform solution; 4) The solution was transferred into a Teflon-lined stainless autoclave, and maintained at 180 °C for 12 hours; 5) As the autoclave cooled down to room temperature, the products were cleaned for several times with DI water and ethanol, and dried at 60 °C for 4 hours under vacuum; 6) The hybrid precursor was annealed at 350 °C for 2 h in order to obtain the CNS/NiCo_2_O_4_ core-shell sub-microspheres.

The phase structures of the samples were characterized by X-ray diffraction spectrometer (XRD) (D8 Advanced XRD; Bruker AXS, Karlsruhe, Germany) with Cu Ka radiation. Raman spectra were carried out by using a Raman spectroscopy (HORIBA Jobin Yvon LabRAM HR). The morphologies of the samples were observed by using a scanning electron microscope (SEM,S-4800; Hitachi High-Technologies Corporation, Japan), a transmission electron microscope (TEM, JEM−2010, JEOL, Japan) and a high resolution transmission electron microscope (HRTEM, JEM 2010FEF, JEOL, Japan). Field emission gun scanning electron microscopy (FEG-SEM) (SEM, Sirion, FEI, Netherlands) with an energy-dispersive X-ray spectrometer (EDS) was employed to characterize the chemical compositions. The surface chemical species of the samples were examined on a X-ray photoelectron spectroscope (XPS, ESCALAB 250Xi, Thermo Fisher Scientific, USA) using Al Ka radiation of 1486.6 eV as the excitation source.

The electrochemical tests were carried out in a 6 M KOH aqueous electrolyte solution at room temperature. The electrochemical properties of the samples were evaluated by using a CHI660D Electrochemical Working Station. All electrochemical measurements were carried out in a three-electrode system, wherein the sample modified nickel foam as the working electrode (WE), platinum as the counter electrode, and saturated calomel electrode (SCE) electrode as the reference electrode. The WE was prepared by mixturing CNS/NiCo_2_O_4_, conductive carbon black and PVDF with a mass ratio of 8:1:1. Then adding appropriate amount of DMF and grinding for one hour to obtain the homogeneous solution. The solution was then casted on nickel foam to obtain an electrode. The assembled electrode pressed at 10 MPa for one minute and dried in a vacuum oven at 60 °C for 12 hours. The mass of active materials coated on each WE is about 1.5 mg.

The specific capacitance (C) was calculated from the slope of each discharge curve, according to the equation C = (I × Δt)/(ΔV × m), where I is the constant discharge current, Δt is the discharge time, ΔV is the voltage difference in discharge and m is the mass of active materials coated on each WE^37^,^38^. Electrochemical impedance spectroscopy (EIS) measurements were made in the frequency range of 0.1–100,000 Hz by applying an AC voltage with 5 mV perturbation.

## Additional Information

**How to cite this article**: Li, D. *et al.* Facile Synthesis of Carbon Nanosphere/NiCo_2_O_4_ Core-shell Sub-microspheres for High Performance Supercapacitor. *Sci. Rep.* **5**, 12903; doi: 10.1038/srep12903 (2015).

## Supplementary Material

Supplementary Information

## Figures and Tables

**Figure 1:**
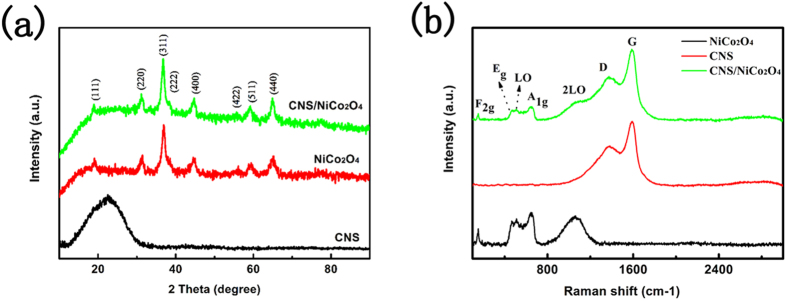
(**a**) XRD patterns of the samples, (**b**) Raman spectra of the samples.

**Figure 2:**
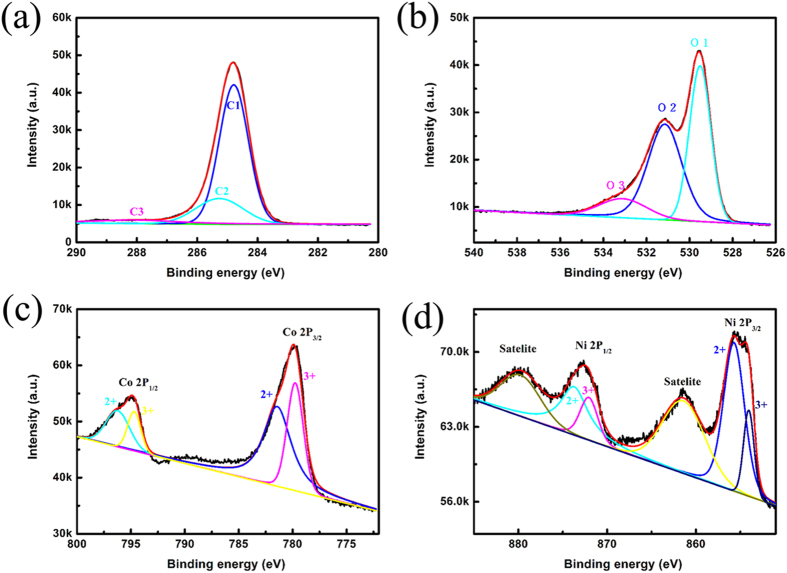
XPS spectra of the CNS/NiCo_2_O_4_ core-shell sub-microspheres: (**a**) C1s, (**b**) O1s, (**c**) Ni 2p, (**d**) Co 2p.

**Figure 3:**
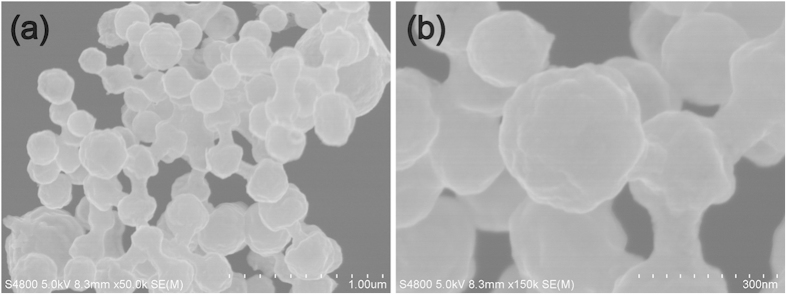
SEM morphology of the CNS/NiCo_2_O_4_ core-shell sub-microspheres: (**a**) low magnification, (**b**) high magnification.

**Figure 4:**
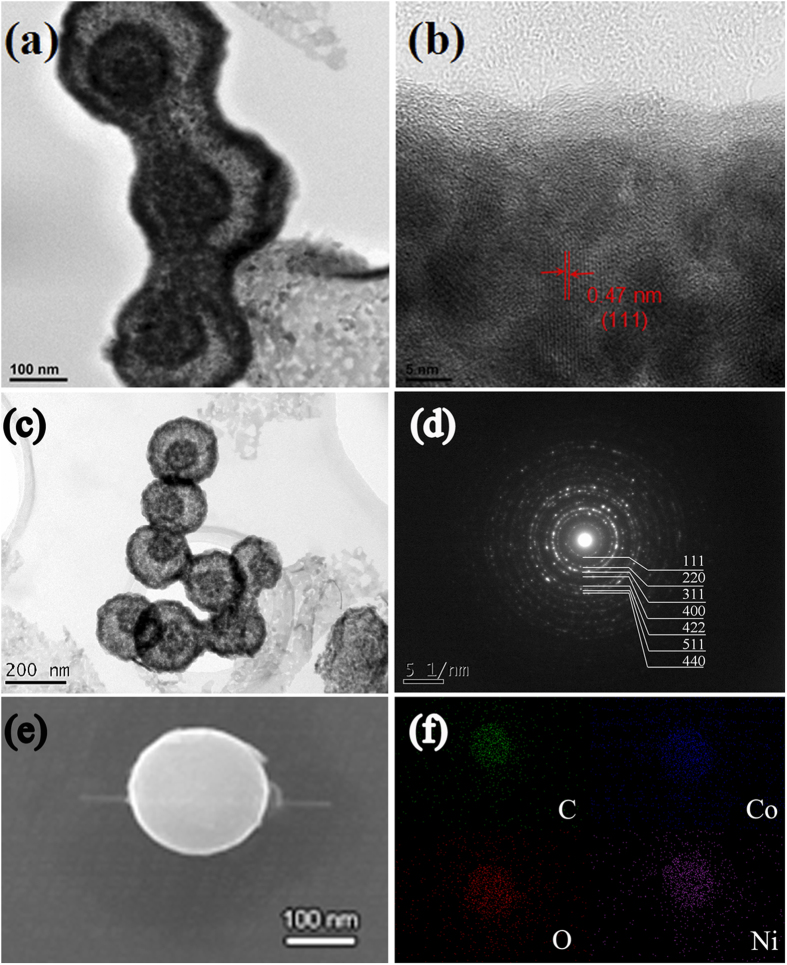
(**a**) low magnification, (**b**) high magnification HRTEM image, (**c**) TEM image, (**d**) corresponds FFT pattern, (**f**) SEM image, and (**e**) element maps of the CNS/NiCo_2_O_4_ core-shell sub-microspheres.

**Figure 5:**
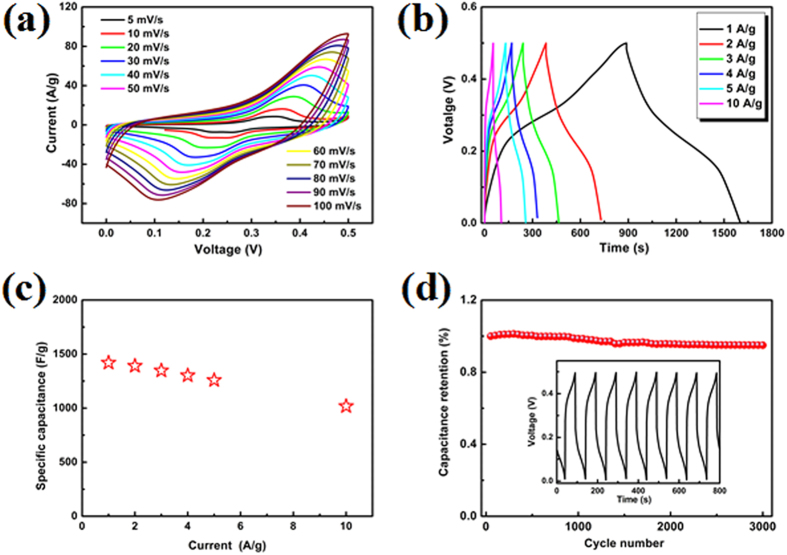
Electrochemical properties of the CNS/NiCo_2_O_4_ core-shell sub-microspheres: (**a**) CV curves at different scan rate, (**b**) GCD curves at different current densities, (**c**) the corresponding specific capacitance as a function of current density, (**d**) cycle performance at current density of 10 A/g, the inset shows charge/discharge curves.

**Figure 6:**
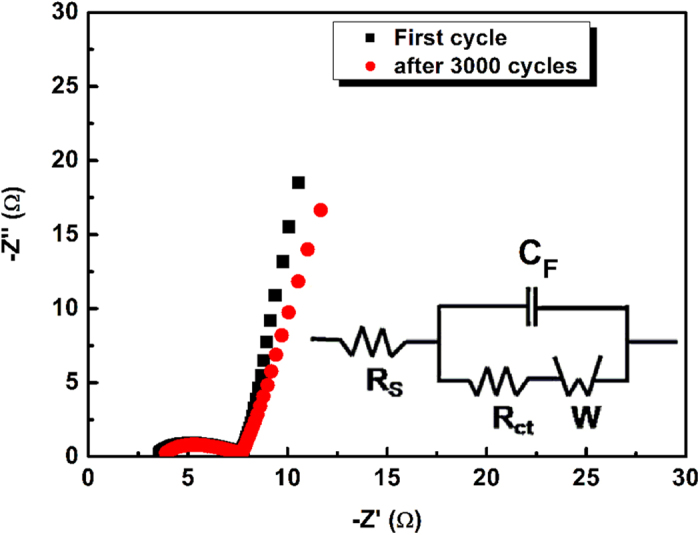
EIS plots of the CNS/NiCo_2_O_4_ modified electrode in 6M KOH solution before and after the cycle test (Inset shows the electrochemical equivalent circuit).
