# Supplementary material for: Facile Synthesis of Carbon Nanosphere/NiCo2O4 Core-shell Sub-microspheres for High Performance Supercapacitor
Source: Sci Rep. 2015 Aug 6;5:12903. doi: 10.1038/srep12903 (PMC4526859; doi:10.1038/srep12903)
Supplement: Supplementary Information [file srep12903-s1.doc]

Supplementary information

**Facile Synthesis of Carbon Nanosphere/NiCo_2_O_4_ Core-shell** **Sub-microspheres for High Performance Supercapacitor**

Delong Li ^a, b^, Youning Gong ^b^, Yupeng Zhang^b, c^, Chengzhi Luo ^b^, Weiping Li ^b^, Qiang Fu^b^, Chunxu Pan ^a, b*^

^a^ Shenzhen Research Institute, Wuhan University, Shenzhen 518057, China.

^b^ School of Physics and Technology, Wuhan University, Wuhan 430072, China.

^c^ Department of Materials Engineering, Monash University, Victoria 3800, Australia

In order to evaluating the content of CNS in the CNS/NiCo_2_O_4_ core-shell Sub-microspheres, TGA was used, as shown in Figure S1. From the thermogravimetry curve, there were two stages during process of mass loss, including removal of adsorbed water and gas before 150ºC and combustion of CNS at about 422ºC. The mass ratio of NiCo_2_O_4_ in the composite is roughly about 5.3%





Figure S1 the TGA –DSC measurement of the CNS/NiCo_2_O_4_ composite in air with a temperature rate of 5K/min

As shown in Figure S2, the CNS/NiCo_2_O_4_ composite had predominant C1s, O1s, Ni2p and Co2p peaks. According to analysis of the XPS spectra, the elemental compositions of the CNS/NiCo_2_O_4_ are list in table S1. The atomic ratio of Ni, Co, and O is 1:1.24: 4.32. The atomic ratio of Ni, Co, and O is not well matching the formula of NiCo_2_O_4_, and the atomic ratio of Co is lower. The Co/Ni atomic ratios (which should be 2 for the stoichiometric compound) show a clear depletion of Co in the surface of the material, which is prepared by thermal decomposition of hybrid precursor (~1.24). The test result is quite familiar with the literature reported^1^. According to Marco and his colleague reported, charge balance when Ni/Co≠0.5 is achieved either by changing the oxidation states of Ni and/or Co surface cations, or by substituting surface O_2_^-^ by hydroxyl groups. However, the atomic ratio of C and O are higher than stoichiometric, which might be effected by the substrate.


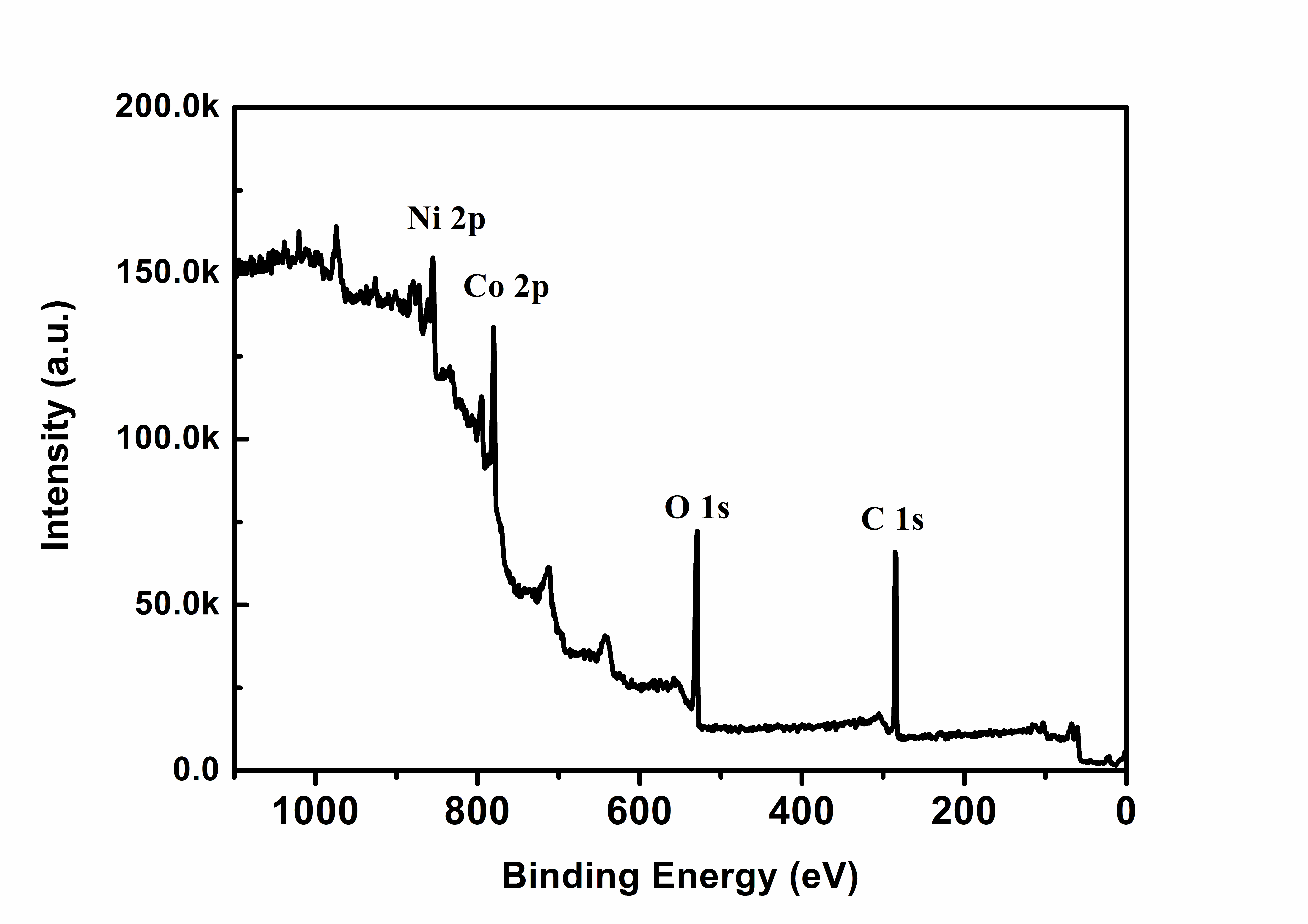


Figure S2. The XPS spectrum of the CNS/NiCo_2_O_4_ composite.

Table S1. The atomic composite ratio of the element

| Element | C | O | Co | Ni |
| --- | --- | --- | --- | --- |
| At% | 53.57 | 30.61 | 8.75 | 7.07 |

Figure S3 shows the EDS pattern of the CNS/NiCo_2_O_4_ composite, and the atomic ratio of the elements are list in Table S2. Obviously, the atomic ratio of Ni, Co, and O is 1:1.97:4.38, which is well matching the formula of NiCo_2_O_4_. A strong Si peak was from the Si substrate.


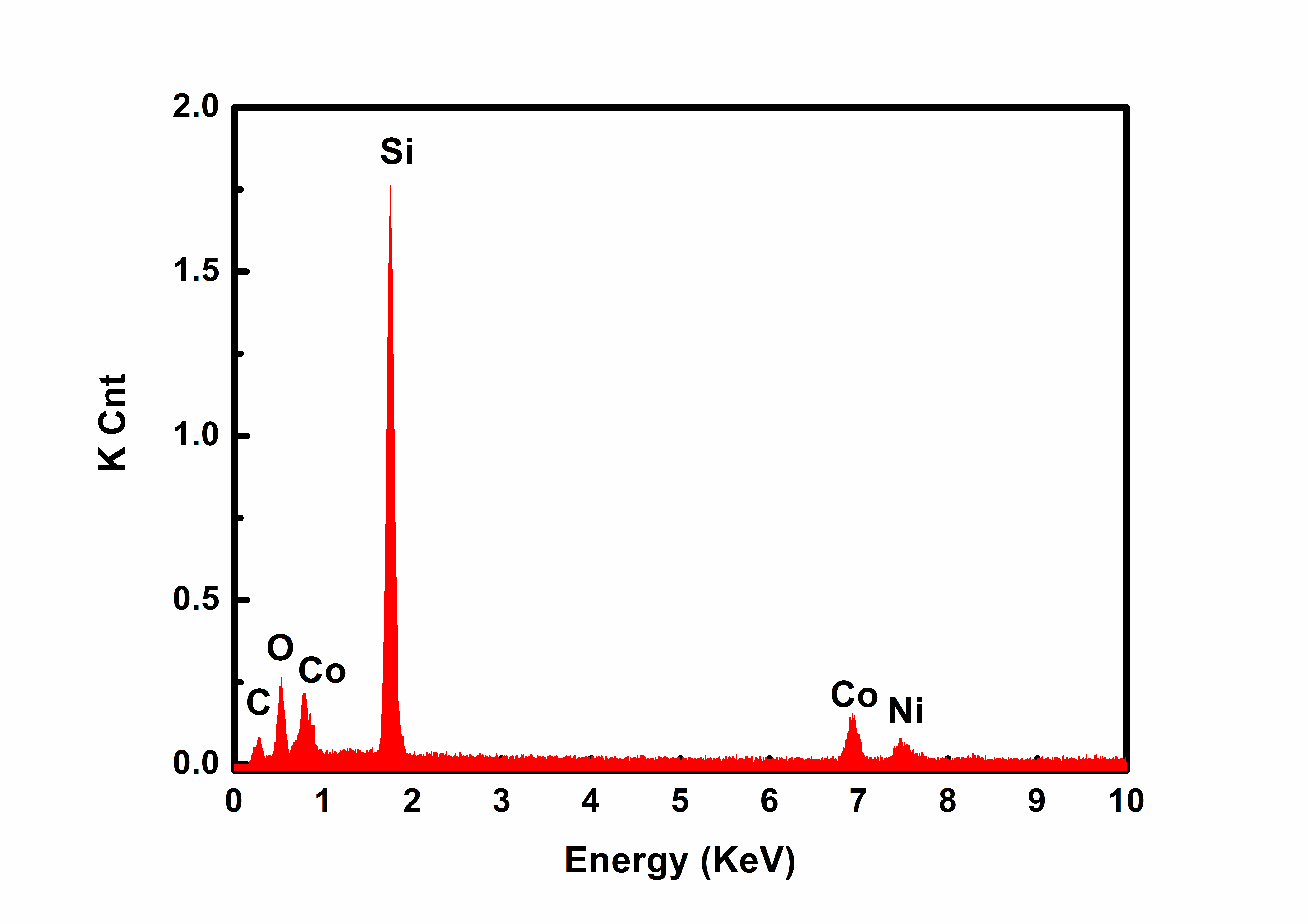


Figure S3. The EDS spectrum of the CNS/NiCo_2_O_4_ composite

Table S2. the atomic ratio of the element

| Element | C | O | Co | Ni |
| --- | --- | --- | --- | --- |
| At% | 27.2 | 43.37 | 19.53 | 9.90 |

Reference

[1] Maco, J. F. et al. Characterization of the nickel cobaltite, NiCo_2_O_4_, prepared by several methods: an XRD, XANES, EXAFS, and XPS study. *J. Solid State Chem.*, **153**, 74-81(2000).
